# Pharmacokinetics of delta-9-tetrahydrocannabinol following acute cannabis smoke exposure in mice; effects of sex, age, and strain

**DOI:** 10.3389/fphar.2023.1227220

**Published:** 2023-08-28

**Authors:** Emely A. Gazarov, Sabrina Zequeira, Alexandria S. Senetra, John Howard, Abhisheak Sharma, Christopher R. McCurdy, Jada Lewis, Jennifer L. Bizon, Barry Setlow

**Affiliations:** ^1^ Department of Neuroscience, University of Florida, Gainesville, FL, United States; ^2^ Department of Psychiatry, University of Florida, Gainesville, FL, United States; ^3^ Department of Pharmaceutics, University of Florida, Gainesville, FL, United States; ^4^ Center for Addiction Research and Education, University of Florida, Gainesville, FL, United States; ^5^ Department of Medicinal Chemistry, University of Florida, Gainesville, FL, United States

**Keywords:** pharmacokinetics, cannabis, Δ9THC, 11-COOH-Δ9THC, smoke

## Abstract

Increased use of cannabis and cannabinoids for recreational and medical purposes has led to a growth in research on their effects in animal models. The majority of this work has employed cannabinoid injections; however, smoking remains the most common route of cannabis consumption. To better model real-world cannabis use, we exposed mice to cannabis smoke to establish the pharmacokinetics of Δ9THC and its metabolites in plasma and brain. To determine the time course of Δ9THC and two major metabolites [11-hydroxy-delta-9-tetrahydrocannabinol (11-OH-THC) and 11-nor-9-carboxy-delta-9-tetrahydrocannabinol (11-COOH-THC)], male and female C57BL/6J mice were exposed to smoke from sequentially burning 5 cannabis cigarettes. Following smoke exposure, trunk blood and brains were collected at 6 time points (10–240 min). Plasma and brain homogenates were analyzed for Δ9THC and metabolites using a validated ultraperformance liquid chromatography-tandem mass spectrometry method. To assess effects of age, sex, and mouse strain, we exposed mice of four strains (C57BL/6J, FVB, Swiss Webster, and 129S6/SvEv, aged 4–24 months) to cannabis using the same smoke regimen. Samples were collected 10 and 40 min following exposure. Lastly, to assess effects of dose, C57BL/6J mice were exposed to smoke from burning 3 or 5 cannabis cigarettes, with samples collected 40 min following exposure. The pharmacokinetic study revealed that maximum plasma Δ9THC concentrations (C_max_) were achieved at 10 and 40 min for males and females, respectively, while C_max_ for brain Δ9THC was observed at 20 and 40 min for males and females, respectively. There were no age or strain differences in plasma Δ9THC concentrations at 10 or 40 min; however, 129S6/SvEv mice had significantly higher brain Δ9THC concentrations than FVB mice. Additionally, 3 cigarettes produced significantly lower plasma 11-COOH-THC concentrations compared to 5 cigarettes, although dose differences were not evident in plasma or brain concentrations of Δ9THC or 11-OH-THC. Across all experiments, females had higher levels of 11-COOH-THC in plasma compared to males. The results reveal robust sex differences in Δ9THC pharmacokinetics, and lay the groundwork for future studies using mice to model the pharmacodynamics of smoked cannabis.

## 1 Introduction

Cannabis is the most widely used illicit drug in the United States, with 18.7% of the population aged 12 or older reporting past year use (Key Substance Use and Mental Health Indicators in the United States: Results from the 2021 National Survey on Drug Use and Health, U.S. Department of Health and Human Services, 2021). With changing attitudes toward cannabis and increasing access to medicinal and recreational markets, there is a critical need for scientific literature to assess the effects of cannabis on human health to inform medical decisions and legal policy making (Hutchison et al., 2019). The increasing accessibility and use of cannabis have also generated interest in understanding its potential therapeutic properties, particularly within the aging population. Various formulations of medical cannabis and cannabis products are currently indicated for the treatment of chronic pain, nausea and vomiting in cancer patients, epilepsy and seizures, and post-traumatic stress disorder (Belendiuk et al., 2015). Additionally, the polypharmaceutical properties of cannabis are of interest for treatment of age-related neurodegenerative conditions such as Alzheimer’s disease, as some evidence suggests that cannabis possesses anti-inflammatory and antioxidant properties (Iuvone et al., 2004; Walter and Stella, 2004; Martín-Moreno et al., 2011; Martín-Moreno et al., 2012). Cannabis-based medications, such as Sativex^®^ and Epidiolex^®^, are already approved for treatment of spasticity in multiple sclerosis patients and epilepsy (Russo, 2018), which encourages further exploration of the effects of cannabis on neurological diseases. With over 90% of genes linked to human diseases also present in the mouse genome (Chinwalla et al., 2002), mice offer a viable option for studying the effects of cannabis and cannabinoids on both general health and specific diseases with translational value to humans. Ease in breeding, relatively short life spans, and the ability to generate transgenic lines also make mice a practical model for studying pharmacological treatments for a variety of disease conditions. When conducting such studies, however, it is important to consider variations in genetic background among mouse strains, as they can have considerable behavioral and physiological differences that alter drug pharmacokinetics. Strain differences in respiratory and cardiac functioning (Reinhard et al., 2002; Campen et al., 2005; Ward et al., 2007; Barnabei et al., 2010), as well as in behaviors such as locomotor activity (Bothe et al., 2004), can all contribute to variability in drug absorption, distribution, and metabolism. Thus, it is important to consider such potential differences in studies of drug pharmacokinetics, particularly in mouse models of disease conditions that may utilize only one or a few background strains.

Inhalation, particularly via smoking, remains the most common route of cannabis use (Knapp et al., 2019; Azcarate et al., 2020). Previous studies have assessed the pharmacokinetic profiles of both injected and inhaled cannabis/cannabinoids in rats by measuring plasma and brain concentrations of Δ9THC (the primary psychoactive component in cannabis) and its two major metabolites, 11-hydroxy-Δ9THC (11-OH-THC) and 11-nor-9-carboxy-Δ9THC (11-COOH-THC) (Hložek et al., 2017; Ruiz et al., 2021a; Ruiz et al., 2021b; Baglot et al., 2021). In mice, however, plasma and brain concentrations of these three analytes have only been thoroughly assessed following injected Δ9THC (Torrens et al., 2020). Inhalation and injections of Δ9THC yield different pharmacokinetic profiles, with differences in distribution, total exposure, and metabolism (Baglot et al., 2021). Such metabolic differences are important to consider, as the primary metabolite of Δ9THC, 11-OH-THC, is also psychoactive and just as potent as Δ9THC, and the secondary metabolite, 11-COOH-THC, remains detectable for 3–7 days following drug intake (Grotenhermen, 2003; Sharma et al., 2012) and may possess anti-inflammatory properties (Ujváry and Grotenhermen, 2014). For those reasons, an increasing number of pharmacological studies of cannabis in rodent models are using an inhaled route of administration, which calls for a thorough assessment of the pharmacokinetic profile of inhaled cannabis in mice. Previous pharmacokinetic studies of inhaled cannabis in mice have been limited to only male mice and/or evaluation of plasma or brain cannabinoids at a limited number of timepoints (Lichtman et al., 2001; Wilson et al., 2006; Poklis et al., 2010; Fantauzzi et al., 2021).

To better characterize the pharmacokinetics of inhaled cannabis in mice, we used a passive cannabis smoke inhalation paradigm to determine how sex, age (young adult, middle-age, advanced age), strain (C57BL/6J, FVB, 129, SW) and dose affect plasma and brain levels of Δ9THC and its metabolites, 11-OH-THC and 11-COOH-THC, across different post-exposure time points.

## 2 Materials and methods

### 2.1 Subjects

Experiment 1 used young adult (3 mo.) male (*n* = 36) and female (*n* = 36) C57BL/6J (B6) mice obtained from the University of Florida Animal Care Services in-house breeding colony (founder mice obtained from The Jackson Laboratories). Experiment 2 used young adult (4–7 mo., *n* = 46), middle aged (10–15 mo., *n* = 37), and aged (20–24 mo., *n* = 7) FVB (Charles River), Swiss Webster (SW) and 129S6/SvEv (Taconic), and C57BL/6J (Jackson Labs) mice. These were non-transgenic mice obtained from in-house transgenic breeding colonies, in keeping with the 3Rs of humane animal practices (Russell and Burch, 1959). These mice were maintained on their respective strain backgrounds for >5 generations. Experiment 3 used young adult (3 mo.) male (*n* = 10) and female (*n* = 10) mice obtained from the same source as Experiment 1. Demographics of the mice in each experiment are described in Table 1. The mice were housed single sex, 1-5 per cage, on a 12-h light/dark cycle (lights on at 0700) with vivarium temperature maintained at 25°C. All experiments took place during the light phase. Water and food (2918 Teklad global 18% protein diet) were provided *ad libitum*. All animal procedures were approved and performed in accordance with the University of Florida Institutional Animal Care and Use Committee and followed National Institutes of Health guidelines.

**TABLE 1:** Mouse demographics.

Experiment 1	(3 months)		
CB7BL/6J	*n* = 72 (36 M, 36 F)		
Experiment 2	Young Adult (4–7 months)	Middle Aged (10–15 months)	Aged (20+ months)
FVB	*n* = 9 (8 M, 1 F)	*n* = 20 (12 M**, 8 F)	
Swiss Webster (SW)	*n* = 14 (6 M*, 8 F)		
129	*n* = 11 (4 M, 7 F)		
C57BL/6J	*n* = 12 (6 M*, 6 F*)	*n* = 17 (10 M#, 7 F*)	n = 7 (2 M, 5 F)
Experiment 3	(3 months)		
C57BL/6J	*n* = 20 (10 M, 10 F)		

Each asterisk (*) represents a single mouse with plasma Δ9THC concentrations outside ±2 SDs from the group mean and removed from analyses.

Each hash mark (#) represents a single mouse with brain Δ9THC concentrations outside ±2 SDs from the group mean and removed from analyses.

### 2.2 Apparatus

A TE-10 automated cigarette smoking machine (Teague Enterprises, Davis, CA United States) was used to conduct smoke exposure sessions. During these sessions, mice remained in their home cages, which were placed into the exposure chamber of the smoking machine (71 × 61 × 61 cm^3^, up to 6 cages containing 1-5 mice each were placed into the exposure chamber simultaneously). Cannabis cigarettes were lit and puffed (35 cm^3^ puff volume, 1 puff per min, 2 s per puff) in the ignition chamber of the smoking machine, from which the smoke (both mainstream and sidestream) was pumped into the exposure chamber via a series of holes on one side of the chamber, and continuously vented out through a series of holes on the opposite side of the chamber and exhausted to the exterior of the building. Once the final cigarette was fully burnt, carbon monoxide (CO) and total suspended particulate matter (TSP) measurements were obtained. The maximum CO level in the exposure chamber (in ppm) was measured using a continuous CO monitor (Monoxor III, Bacharach, New Kensington, PA USA). For this measurement, air was pumped out of the exposure chamber and into the monitor for 2 minutes. TSP measurements were obtained by pumping air out of the exposure chamber and passing it through a pre-weighed filter (United Filtration Systems Inc, Sterling Heights, MI USA) for 2 minutes. To calculate the TSP (mg/cubic meter), the total weight gained by the filter was divided by the volume of airflow passing through the filter, which was measured by a dry gas meter. In Experiment 1, the average TSP level was 78 ± 12 mg/m^3^ and the CO level was 130 ± 27 ppm. In Experiment 2, the average TSP level was 89 ± 23 mg/m^3^ and the CO level was 101 ± 13 ppm. In Experiment 3, the 3-cigarette condition produced a TSP level of 125 mg/m^3^ and CO level of 201 ppm, and the 5-cigarette condition produced a TSP level of 175 mg/m^3^ and CO level of 209 ppm.

### 2.3 Experimental design

Mice were exposed to smoke generated from sequentially burning cannabis cigarettes (NIDA Drug Supply Program, approximately 700 mg each) containing 5.5%–6.2% THC (∼40 mg THC per cigarette) and less than 0.01% CBD (Bruijnzeel et al., 2016; Bruijnzeel et al., 2019). In Experiment 1, mice were exposed to smoke from sequentially burning 5 cannabis cigarettes in 1 h (approximately 12 min per cigarette), after which they were removed from the exposure chamber. Upon removal, mice of each sex were euthanized at 10, 20, 40, 60, 120, and 240 min time points post-smoke exposure (mice from different cages were used at each time point). In Experiment 2, mice followed the same smoke regimen as Experiment 1; however, they were euthanized only at 10 and 40 min time points post-smoke exposure (roughly the times of peak Δ9THC and metabolite concentrations in plasma and brain) to evaluate strain and age differences in these measures. In Experiment 3, mice were exposed to smoke from burning either 3 cannabis cigarettes in 36 min or 5 cannabis cigarettes in 1 h, in order to determine cannabinoid levels following different durations (doses) of cannabis smoke exposure. Because cannabis cigarettes are a limited resource, the objective of this last experiment was to determine if smoking duration could be reduced without compromising Δ9THC plasma and brain concentrations in future studies involving chronic smoke exposure. In Experiment 3, all mice were euthanized 40 min post-smoke exposure to allow for comparisons at a time point at which peak brain Δ9THC concentrations are achieved and plasma Δ9THC concentrations remain well within the detectible range.

### 2.4 Sample collection

Mice were euthanized via rapid decapitation at specific time points following smoke exposure, with 0 min defined as the time at which the cages were removed from the exposure chamber. Trunk blood was collected from each mouse using MicrovetteCB 300 LH 0.3 mL tubes (Sarstedt Inc, Newton, NC United States), which were temporarily stored on ice. Brains were harvested and flash frozen for 30 s by submerging them in 2-methylbutane chilled in a dry ice/ethanol bath, followed by placement in a sealed tube and temporary storage in dry ice. In Experiment 2, tail clippings were collected for genotyping and temporarily stored in ice. All blood samples were centrifuged for 15 min at 4°C at 6,500 rpm (∼4,000 x g) to separate plasma from blood. Plasma was pipetted from each sample and transferred to a polypropylene 0.6 mL microcentrifuge tube. All plasma and brain samples were stored at −80°C until analysis. Tail clippings were stored at −20°C until genotyping.

The mice obtained from internal transgenic breeding colonies used in Experiment 2 were genotyped both when weaned and when euthanized to confirm non-transgenic/wild-type status, based on the colony from which they were derived. Mice on the FVB background were genotyped for either the human tau transgene (Santacruz et al., 2005) by Transnetyx or internally by PCR for the human matrin transgene (Moloney et al., 2018). Mice on the 129 background were genotyped by Transnetyx for the tTA transgene (Santacruz et al., 2005). Mice on the SW background were genotyped for the human tau transgene (Lewis et al., 2000) by Transnetyx. Mice on the B6 background were genotyped for the presence of a progranulin knock-out allele (Ahmed et al., 2010), ATP13A2 knock-out allele (Rayaprolu et al., 2018), or the NEFH-tTA transgene (Walker et al., 2015) by PCR, the latter according to the protocol published on the Jackson Laboratories website (https://www.jax.org/Protocol?stockNumber=025397&protocolID=18061).

### 2.5 Chemicals and reagents

Commercially available standards for delta-8-tetrahydrocannabinol (Δ8THC) (100 μg/mL), Δ9THC (100 μg/mL), 11-OH-THC (100 μg/mL), 11-COOH-THC (100 μg/mL), and delta-9-tetrahydrocannabinol-d3 (Δ9THC-d3) [(100 μg/mL); internal standard (IS)] stock solutions were obtained from Cerilliant (Round Rock, TX, United States). Liquid chromatography-mass spectrometry (LC-MS) grade water, methanol, and formic acid were purchased from Fisher Scientific (Fair Lawn, NJ, United States).

### 2.6 UPLC-MS/MS analysis

Primary stock solutions were diluted to obtain secondary mix stocks of 1,000 ng/mL and 10,000 ng/mL of Δ8THC, Δ9THC, 11-OH-THC, and 11-COOH-THC, in acetonitrile. These stocks were then further diluted in acetonitrile to produce working stocks for calibration standards (CS) of 25, 50, 100, 250, 500, 1,000, 1,500, and 2,500 ng/mL of Δ8THC, Δ9THC, 11-OH-THC, and 11-COOH-THC. Quality control (QC) working stock solutions were prepared from a second set of stocks at concentrations of 25, 75, 1,250, and 2000 ng/mL.

A Waters Acquity Class-I UPLC coupled with a Xevo TQ-S Micro triple quadrupole mass spectrometer (Waters, Milford, MA, United States) was used for the quantitative analysis. An in-house bioanalytical method available for the simultaneous quantification of Δ8THC, Δ9THC, 11-OH-THC, and 11-COOH-THC was translated and validated for quantitative analysis in plasma and brain homogenates (Penman et al., 2023). A gradient method was applied to achieve chromatographic separation using a mobile phase consisting of water containing 0.1% formic acid (A) and methanol (B) and a Waters Acquity BEH C18 column (1.7 μm, 2.1 × 100 mm) at a flow rate of 0.35 mL/min. The 5-min method started at a gradient of 25% A until 0.5 min then decreased to 5% until 4.5 min then re-equilibrated to 25% A until 5 min. Ionization of Δ8THC, Δ9THC, 11-OH-THC, and 11-COOH-THC was achieved in positive mode using electrospray ionization (ESI). The mass spectral analysis was achieved by multiple reaction monitoring (MRM), and the compound parameters for analytes and IS are described in Table 2.

**TABLE 2:** Compound parameters for analytes and internal standard (IS).

Compound	Mass transition (*m/z*)	Cone voltage (V)	Collision energy (V)	Retention time (min)
delta-8-tetrahydrocannabinol	315.24 > 122.99	26	34	3.44
delta-9-tetrahydrocannabinol	315.24 > 123.01	4	40	3.33
11-hydroxy-delta-9-tetrahydrocannabinol	331.25 > 193.11	6	22	2.10
11-nor-9-carboxy-delta-9-tetrahydrocannabinol	345.23 > 193.18	2	26	2.29
delta-9-tetrahydrocannabinol-D_3_ (IS)	318.30 > 196.09	58	24	3.31

Drug-free plasma or brain homogenate was used to prepare CS and QC samples containing Δ8THC, Δ9THC, 11-OH-THC, and 11-COOH-THC. Prior to analysis, each brain was weighed and homogenized in triple distilled water at a ratio of one part brain to three parts water. CSs were generated by spiking 18 μL blank plasma or brain homogenate with 2 μL of calibration mix stocks to get a linear range of 2.5, 5, 10, 25, 50, 100, 150, and 250 ng/mL. The same process was used to generate the lower limit of quantification (LLOQ, 2.5 ng/mL) and low (LQC, 7.5 ng/mL), medium (MQC, 125 ng/mL), and high (HQC 200 ng/mL) QCs. After spiking, the CS and QC samples were vortex-mixed for 5 min at 650 rpm to ensure homogeneity. To prepare samples for analysis, 20 μL of plasma or brain homogenate samples, along with respective CS and QC samples, were aliquoted and quenched with 80 μL (1:4 ratio) of methanol containing 0.05% v/v formic acid and IS (25 ng/mL) to precipitate proteins. The quenched samples were vortex-mixed for 5 min at 650 rpm and then filtered through a 0.45 µm filter plate (Millipore, Burlington, MA, United States) for 3 min at 1,500 rpm at 4°C. The filtrates of all samples were then subjected to UPLC-MS/MS analysis.

A validated bioanalytical method, available in mouse serum, was further partially validated for sensitivity, selectivity, linearity, carryover, accuracy, and precision for the quantification of Δ8THC, Δ9THC, 11-OH-THC, and 11-COOH-THC in both mouse plasma and brain homogenate following Food and Drug Administration (FDA) guidelines for bioanalytical method validation (FDA, 2018). The LOD for each analyte was found at 1 ng/mL (response signal-to-noise ratio >3), while the LLOQ was selected at 2.5 ng/mL for all analytes (signal-to-noise ratio >10) with accuracy and precision within 20%. There were no endogenous substances eluting at the retention time of any analyte or IS when analyzing blank plasma or brain homogenate (Supplementary Figure S1). The calibration curve had a concentration range of 2.5–250 ng/mL and was found to be linear for all analytes with a coefficient of determination value > 0.99 for all runs. Carryover analysis of a blank sample immediately following an HQC of 200 ng/mL produced an analyte peak area <20% of the LLOQ for all analytes, and <5% of IS, showing negligible carryover. Intra- and inter-day accuracy and precision were performed during three different days at each QC level for both plasma and brain homogenate (Supplementary Table S1). All values were within acceptable limits of 15% target concentration, and within 20% for LLOQ concentration.

The TargetLynx™ application of MassLynx™ 4.2 was used for data processing and quantification of the UPLC/MS-MS data (Waters, Milford, MA, United States). Phoenix Version 6.4 (Certara, Princeton, NJ, United States) was used for the non-compartmental analysis of concentration-time data.

### 2.7 Statistical analysis

Statistical analyses were conducted using SPSS 27.0 (IBM, Armonk, NY, United States) and graphs were constructed in GraphPad Prism 9 (GraphPad Software, San Diego, CA, United States). In Experiment 1, plasma and brain Δ9THC and metabolite concentrations were analyzed using two-factor ANOVA, with sex (2 levels) and time post-smoke exposure (6 levels) as between-subjects factors, to assess sex differences and the effect of time following smoke exposure. In Experiment 2, strain comparisons between young adult mice were analyzed using a two-factor ANOVA, with strain (4 levels) and time (2 levels) as between-subjects factors. Significant effects of strain were further explored using a Tukey *post hoc* analysis. Additionally, the effect of sex was evaluated across strains in young adult mice using a two-factor ANOVA, with sex (2 levels) and time (2 levels) as factors. For age comparisons in mice of the B6 and FVB strains, data were analyzed using two-factor ANOVA, with time (2 levels) and age group (3 levels for B6 mice and 2 levels for FVB mice) as factors. Six Δ9THC plasma concentrations and one Δ9THC brain concentration were statistical outliers (greater than ±2 SDs outside the group mean) and removed from analyses. Demographics of outliers are described in Table 1. In Experiment 3, the effects of smoke exposure duration on plasma and brain Δ9THC and metabolite concentrations were analyzed using two-factor ANOVA, with the number of cannabis cigarettes (2 levels) and sex (2 levels) as factors. Significance was defined as *p* < 0.05 for all analyses.

## 3 Results

### 3.1 Experiment 1. Pharmacokinetics of Δ9THC and its metabolites following cannabis smoke exposure

**FIGURE 1:**
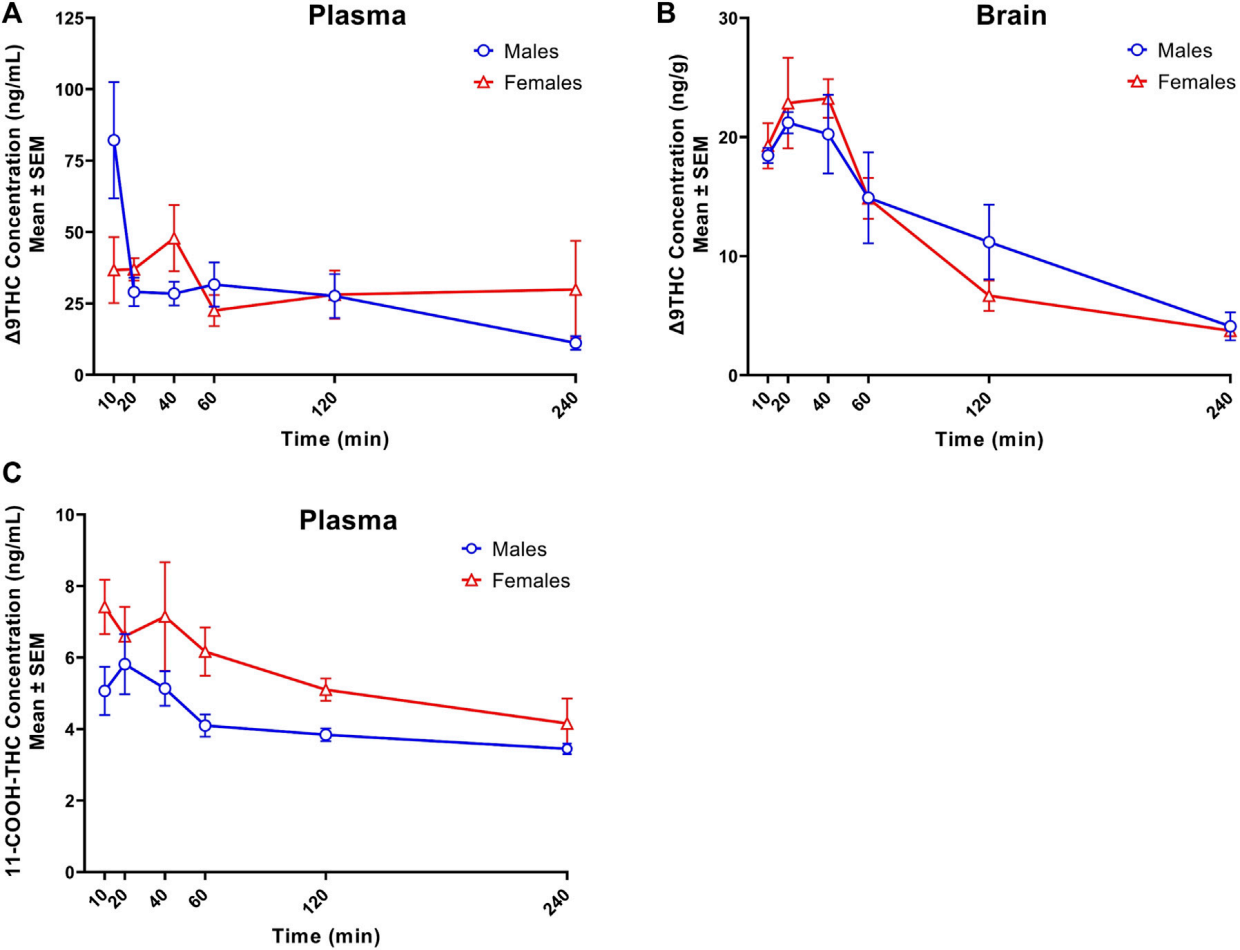
Concentration-time profiles of Δ9THC and 11-COOH-THC following 1 h cannabis smoke exposure in young adult C57BL/6J mice (Experiment 1). Maximum plasma Δ9THC concentrations were achieved 10 and 40 min post-smoke exposure in males (C_max_ = 82.2 ng/mL) and females (C_max_ = 47.9 ng/mL), respectively. A significant Sex × Time interaction was observed in plasma Δ9THC concentrations, with males having greater concentrations 10 min post-smoke exposure compared to females **(A)**. Maximum brain Δ9THC concentrations were achieved 20- and 40-min post-smoke exposure in males (C_max_ = 21.2 ng/g) and females (C_max_ = 23.2 ng/g), respectively. There was a significant main effect of Time on brain Δ9THC concentrations, with levels declining after peak concentrations were achieved **(B)**. A significant sex difference was observed in plasma 11-COOH-THC concentrations, with females achieving higher concentrations than males **(C)**. Data are represented as the mean of individual mouse analyte concentrations at each time point.

Figure 1 displays plasma Δ9THC, brain Δ9THC, and plasma 11-COOH-THC concentration-time profiles following 1 h exposure to cannabis smoke. The experiment was conducted in two cohorts for a total of 72 B6 mice (half female in each cohort). Δ9THC and metabolite concentrations from both cohorts were combined and averaged for each time point. Plasma and brain Δ8THC concentrations were below the LLOQ (2.5 ng/mL or ng/g) in samples collected from all 72 mice (as well as in Experiments 2 and 3), and will not be described further. The pharmacokinetic profile of Δ9THC in brain and plasma after inhalation is described in Table 3.

**TABLE 3:** Pharmacokinetic parameters of Δ9THC in brain and plasma after inhalation (Exp 1).

Parameter	Brain			Plasma		
	Combined^a^	Male^b^	Female^c^	Combined^a^	Male^b^	Female^c^
**Dose (mg/kg)**	0.19	0.18	0.19	0.19	0.18	0.19
**C**_**max**_ **(ng/mL or ng/g)**	22.0	21.2	23.2	70.4 ± 9.7^d^	82.2	47.9
**T**_**max**_ **(h)**	0.33	0.33	0.67	0.73 ± 0.27^d^	0.17	0.67
**AUC**_**last**_ **(h×ng/mL or h×ng/g)**	44.7	47.5	41.9	120.2	123.5	121.4
**Cl/F (L/h/kg)**	-	-	-	0.7	1.2	0.3
**V**_**z**_ **/F(L/kg)**	-	-	-	5.1	3.2	5.3

^a^ Values represent the average of all samples at each time point (male and female mice).

^b^ Values represent the average of all male samples at each time point.

^c^ Values represent the average of all female samples at each time point.

^d^ Values represent the mean of all samples ±SEM.

Abbreviations: AUC_last_ = area under the plasma concentration-time profile up to the last measurable concentration, Cl = clearance, C_max_ = maximum plasma concentration after exposure, T_max_ = time to reach C_max_, V_z_ = volume of distribution, F = bioavailability fraction.

Peak plasma Δ9THC concentrations (C_max_) in male and female mice were achieved at 10 min and 40 min, respectively, after smoke exposure, with males achieving a comparatively higher C_max_ than females (C_max_ males = 82.2 ng/mL, C_max_ females = 47.9 ng/mL; Figure 1A). Consistent with this difference in timing and magnitude of C_max_, a two-way ANOVA (Time x Sex) revealed a significant Time × Sex interaction (F (5,58) = 2.854, *p* = 0.023) in addition to a main effect of Time, such that plasma Δ9THC levels declined across time points (F (5,58) = 3.595, *p* = 0.007), although there was no main effect of Sex (F (1,58) = 0.053, *p* = 0.818). The measured plasma Δ9THC concentration-time data were subjected to linear trapezoidal method and an area under the curve (AUC) of 123.5 and 121.4 h x ng/mL was observed in male and female mice, respectively.

Brain Δ9THC C_max_ values in male and female mice were achieved at 20 and 40 min, respectively, after smoke exposure (C_max_ males = 21.2 ng/g, C_max_ females = 23.2 ng/g; Figure 1B). A two-way ANOVA (Time x Sex) revealed a main effect of Time (F (5,58) = 18.055, *p* < 0.0001), such that brain Δ9THC concentrations declined across time points, but neither a main effect of Sex (F (1,58) = 0.004, *p* = 0.947) nor a Time × Sex interaction (F (5,58) = 0.606, *p* = 0.696). The measured brain Δ9THC concentration-time data were subjected to linear trapezoidal method and an AUC of 47.5 and 41.9 h x ng/g was observed in male and female mice, respectively.

Males achieved peak plasma 11-OH-THC concentrations (3.45 ng/mL) 20 min post-smoke exposure and females achieved peak concentrations (3.97 ng/mL) 10 min post-smoke exposure (Table 4). Following peak plasma 11-OH-THC concentrations in both males and females, however, a majority of samples (≥5 mice per sex per time point) were below the LLOQ. Males achieved peak brain 11-OH-THC concentrations (5.93 ng/mL) 10 min following exposure, whereas females achieved peak concentration (3.9 ng/mL) 40 min post exposure. 11-OH-THC concentrations in brain samples collected beyond the 40 min time point were largely below the LLOQ (Table 5).

**TABLE 4:** Time course of plasma Δ9THC, 11-OH-THC, and 11-COOH-THC concentrations.

Exp. 1	Plasma Concentrations Males			Plasma Concentrations Females		
Time (min)	Δ9THC (ng/mL)	11-OH-THC (ng/mL)	11-COOH-THC (ng/mL)	Δ9THC (ng/mL)	11-OH-THC (ng/mL)	11-COOH-THC (ng/mL)
**10**	82.2 ± 20.4	3.4 ± 0.5	5.1 ± 0.7	36.7 ± 11.6	4.0 ± 0.9	7.4 ± 0.8
**20**	29.1 ± 5.0	3.5 ± 0.3	5.8 ± 0.8	37.0 ± 3.9	< LLOQ	6.6 ± 0.8
**40**	28.5 ± 4.1	< LLOQ	5.1 ± 0.5	47.9 ± 11.6	< LLOQ	7.2 ± 1.5
**60**	31.6 ± 7.8	< LLOQ	4.1 ± 0.3	22.6 ± 5.5	< LLOQ	6.2 ± 0.7
**120**	27.6 ± 7.7	< LLOQ	3.8 ± 0.2	28.1 ± 8.5	< LLOQ	5.1 ± 0.3
**240**	11.2 ± 2.4	< LLOQ	3.5 ± 0.2	29.9 ± 15.5	< LLOQ	4.2 ± 0.7

Abbreviations: LLOQ, lower limit of quantification.

**TABLE 5:** Time course of brain Δ9THC, 11-OH-THC, and 11-COOH-THC concentrations.

Exp. 1	Brain Concentrations Males			Brain Concentrations Females		
Time (min)	Δ9THC (ng/g)	11-OH-THC (ng/g)	11-COOH-THC (ng/g)	Δ9THC (ng/g)	11-OH-THC (ng/g)	11-COOH-THC (ng/g)
**10**	18.5 ± 0.6	5.9 ± 1.1	< LLOQ	19.3 ± 1.9	3.1 ± 0.2	< LLOQ
**20**	21.2 ± 0.9	3.4 ± 0.4	< LLOQ	22.9 ± 3.8	< LLOQ	< LLOQ
**40**	20.2 ± 3.3	3.6 ± 0.4	< LLOQ	23.2 ± 1.6	3.9 ± 0.6	< LLOQ
**60**	14.9 ± 3.8	< LLOQ	< LLOQ	14.9 ± 1.7	< LLOQ	< LLOQ
**120**	11.2 ± 3.1	< LLOQ	< LLOQ	6.7 ± 1.3	< LLOQ	< LLOQ
**240**	4.1 ± 1.2	< LLOQ	< LLOQ	3.7 ± 0.5	< LLOQ	< LLOQ

Abbreviations: LLOQ, lower limit of quantification.

In contrast to plasma 11-OH-THC, concentrations of the secondary Δ9THC metabolite 11-COOH-THC were above the LLOQ in all but six plasma samples. Of those six samples below the LLOQ, five were from males at the 120 and 240 min time points. A two-way ANOVA (Time x Sex) revealed a significant sex difference in plasma 11-COOH-THC concentrations (F (1,54) = 10.605, *p* = 0.002, Figure 1C) with females maintaining higher concentrations than males at all 6 time points. Additionally, there was a main effect of Time (F (5, 54) = 2.845, *p* = 0.024) such that concentrations declined across time points, but no Time × Sex interaction (F (5,54) = 0.380, *p* = 0.860). Brain 11-COOH-THC concentrations were below the LLOQ in samples from all 72 mice.

### 3.2 Experiment 2. Δ9THC and its metabolites following cannabis smoke exposure in mice of different strains and ages

Non-transgenic mice of four different strains (n = 90) used commonly to study neurodegenerative and other neurological conditions were exposed to smoke from burning 5 cannabis cigarettes sequentially, and plasma and brain samples were collected 10 and 40 min after exposure. In two of the strains, mice of different ages (young adult, middle-aged, advanced age) were used to determine whether age affects Δ9THC pharmacokinetics. Because limitations in availability resulted in mice of both sexes and all ages not being represented equally across all strains and post-exposure time points (see Table 1), separate analyses were used to a) compare strains in young adults; b) compare males and females in young adult and middle-aged mice; and c) compare age groups in the B6 and FVB strains. One mouse was excluded from the analyses due to positive transgenic status (female B6 mouse). Brain concentrations of 11-OH-THC and 11-COOH-THC were below the LLOQ for all samples. Plasma 11-OH-THC concentrations were above the LLOQ for 51/90 samples; however, there was an unequal distribution of quantifiable samples across strains and ages.

#### 3.2.1 Effects of strain on Δ9THC and its metabolites

**FIGURE 2:**
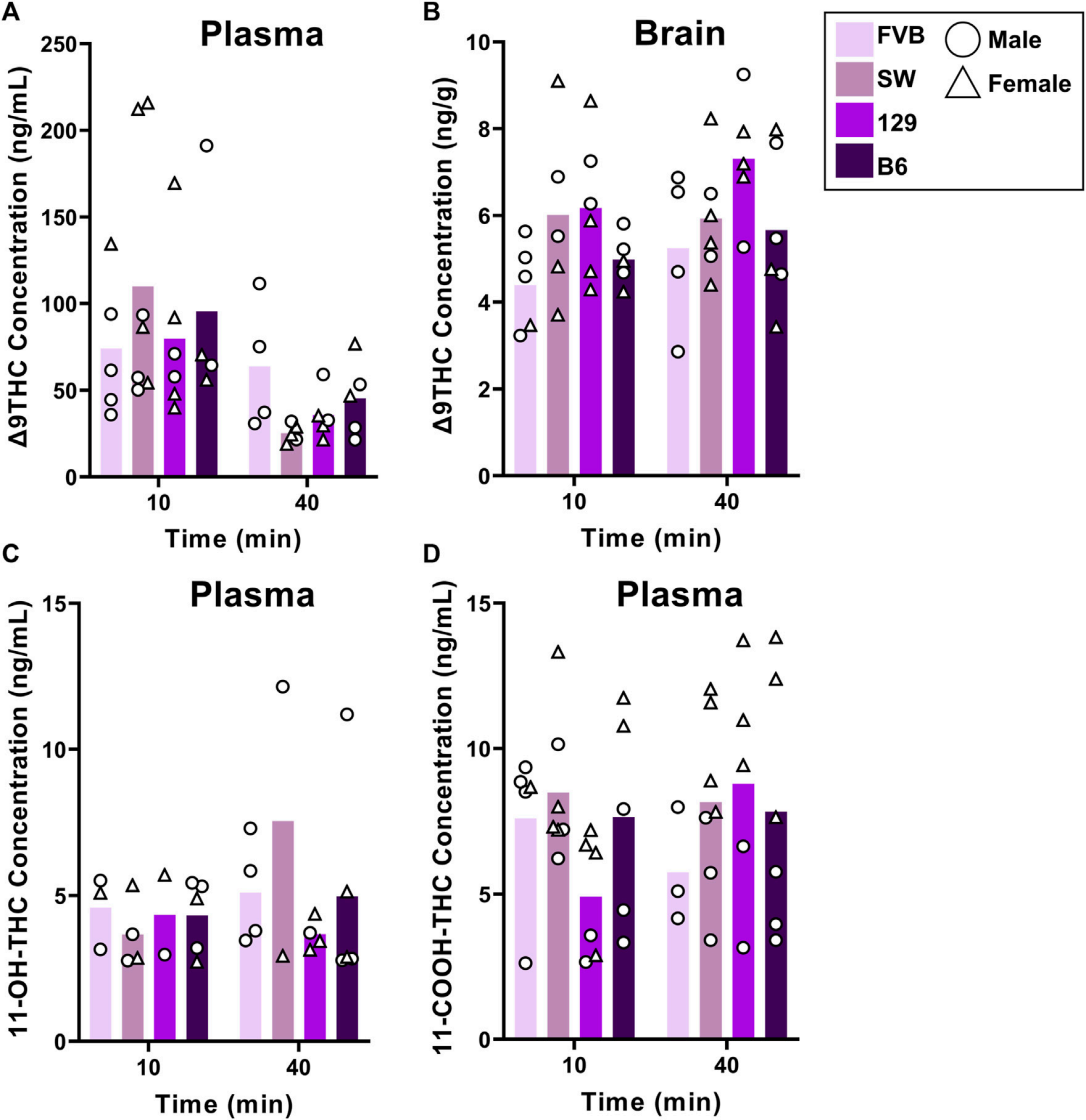
Effects of strain on Δ9THC and its metabolite concentrations after smoke exposure in young adult mice (Experiment 2). No strain differences were observed in plasma Δ9THC concentrations **(A)**. There was a significant main effect of Strain on brain Δ9THC concentrations, with 129 mice achieving significantly greater concentrations than FVB mice **(B)**. A main effect of strain was not observed in plasma 11-OH-THC **(C)** or plasma 11-COOH-THC concentrations **(D)**.

Figure 2 shows plasma and brain Δ9THC and plasma metabolite concentrations in young adult mice of all four strains following cannabis smoke exposure. Assessment of plasma Δ9THC concentrations using a two-way ANOVA (Time x Strain) revealed a significant main effect of Time (F (1,33) = 7.230, *p* = 0.003), such that, as expected, concentrations were lower at 40 than 10 min post-exposure (Figure 2A); however, there was no main effect of Strain (F (3,33) = 0.164, *p* = 0.920) or Strain × Time interaction (F (3,33) = 1.132, *p* = 0.351). In contrast, the same analysis conducted on brain Δ9THC concentrations revealed neither a main effect of Time (F (1,34) = 1.832, *p* = 0.185) nor a Strain × Time interaction (F (3,34) = 0.310, *p* = 0.818), but there was a significant effect of Strain (F (3,34) = 2.972, *p* = 0.045, Figure 2B). Tukey *post hoc* comparisons revealed that 129 mice had significantly higher brain Δ9THC concentrations than FVB mice (*p* = 0.041), but that no other comparisons reached statistical significance. For the primary (11-OH-THC) and secondary (11-COOH-THC) metabolites in plasma, there were neither significant main effects of Strain and Time nor an interaction between the two variables (Fs < 1.428, ps > 0.250; Figures 2C, D).

#### 3.2.2 Effects of sex on Δ9THC and its metabolites

**FIGURE 3:**
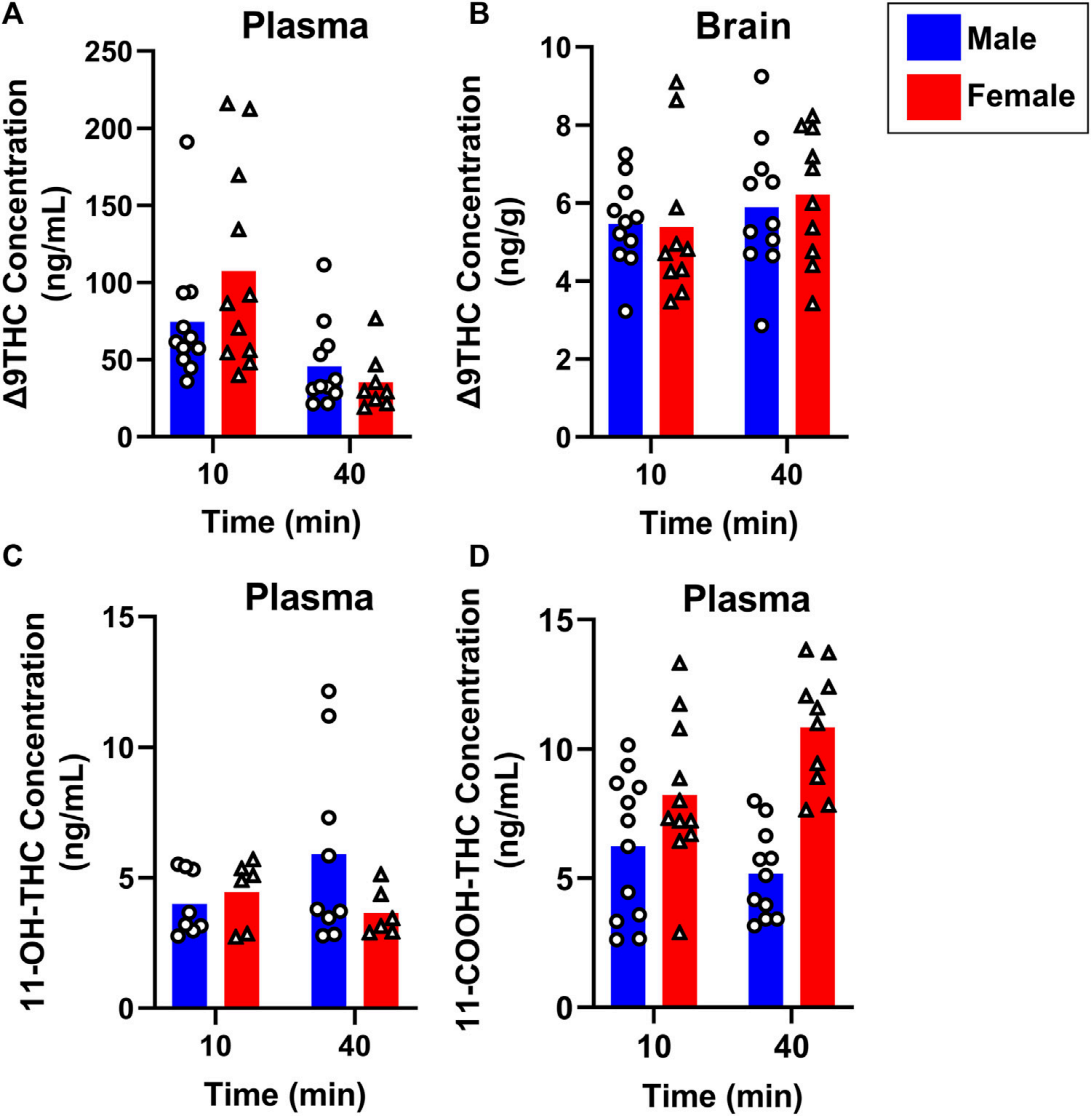
Effects of sex on Δ9THC and its metabolite concentrations after smoke exposure in young adult mice (Experiment 2). Collapsed across strains, there was no significant effect of Sex on plasma Δ9THC **(A)**, brain Δ9THC **(B)**, or plasma 11-OH-THC **(C)** concentrations. Significant sex differences were observed in plasma 11-COOH-THC concentrations, with females achieving greater levels than males **(D)**.

**FIGURE 4:**
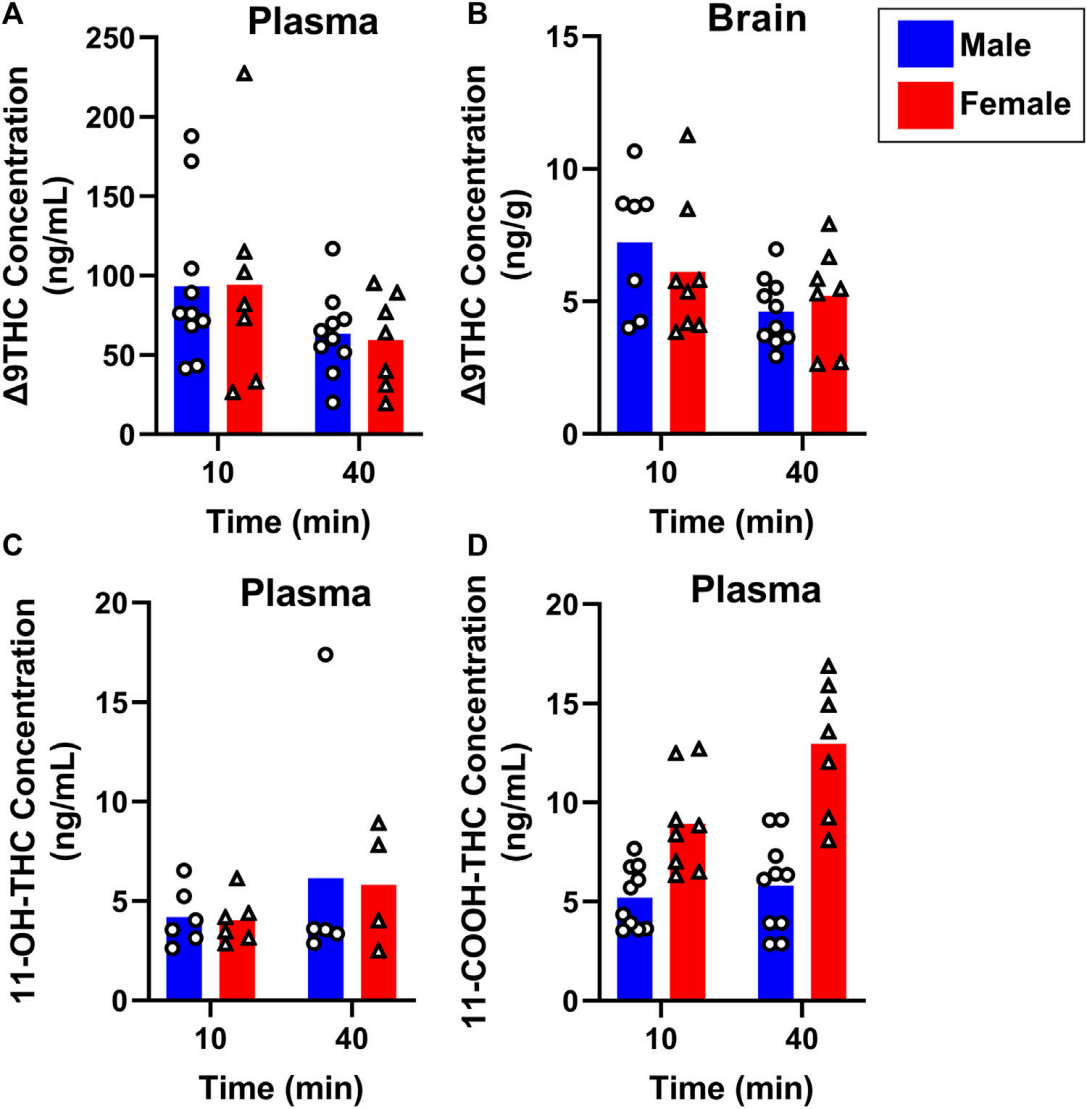
Effects of sex on Δ9THC and its metabolite concentrations after smoke exposure in middle-aged mice (Experiment 2). Collapsed across strains, there was no significant effect of Sex on plasma Δ9THC **(A)**, brain Δ9THC **(B)**, or plasma 11-OH-THC **(C)** concentrations. Significant sex differences were observed in plasma 11-COOH-THC concentrations, with females achieving greater levels than males **(D)**.

To evaluate sex differences in Δ9THC plasma and brain concentrations in young adult mice, data were collapsed across strain and evaluated via a two-factor ANOVA (Sex x Time). For Δ9THC plasma concentrations (Figure 3A), there was a significant main effect of Time (F (1,37) = 13.285, *p* < 0.001) such that mice of both sexes had lower Δ9THC concentrations 40 min after smoke exposure compared to 10 min after exposure, but neither a main effect of Sex (F (1,37) = 0.639, *p* = 0.429) nor a Sex × Time interaction (F (1,37) = 2.421, *p* = 0.128). For brain Δ9THC concentrations (Figure 3B), there were neither main effects of Sex (F (1,38) = 0.056, *p* = 0.815) nor Time (F (1,38) = 1.560, *p* = 0.219), nor was there a Sex × Time interaction (F (1,38) = 0.162, *p* = 0.690). The same analyses conducted in middle aged mice (collapsed across FVB and B6 strains only), revealed patterns similar to those in young adults for Δ9THC plasma concentrations (Figure 4A), with a significant main effect of Time (F (1,30) = 4.238, *p* = 0.048) but no main effect of Sex (F (1,30) = 0.008, *p* = 0.927) and no Sex × Time interaction (F (1,30) = 0.026, *p* = 0.872). There were also no sex differences in Δ9THC brain concentrations in middle aged mice (F (1,28) = 0.127, *p* = 0.724, Figure 4B) nor was there a Sex × Time interaction (F (1,28) = 1.352, *p* = 0.255), but there was a significant main effect of Time (F (1,28) = 5.489, *p* = 0.026), with middle aged mice of both sexes having lower concentrations at 40 compared to 10 min after smoke exposure.

Sex differences in plasma primary and secondary Δ9THC metabolite concentrations were also assessed in young adult mice, collapsed across strains. For 11-OH-THC, there were neither main effects of Sex (F (1,25) = 1.140, *p* = 0.296) nor Time (F (1,25) = 0.427, *p* = 0.520), nor a Sex × Time interaction (F (1,25) = 2.508, *p* = 0.126; Figure 3C). In contrast, for plasma 11-COOH-THC, although there was no main effect of Time (F (1,40) = 1.102, *p* = 0.300), there was a significant main effect of Sex (F (1,40) = 26.386, *p* < 0.001) as well as a Sex × Time interaction (F (1,40) = 6.046, *p* = 0.018) with the greater levels in females compared to males being more robust at 40 compared to 10 min (Figure 3D). Among middle-aged mice (collapsed across FVB and B6 strains only), the patterns of results were comparable to those observed in young mice, with no main effects or interactions for plasma 11-OH-THC (Figure 4C) but a significant main effect of Sex (F (1,31) = 43.097, *p* < 0.001) and Sex × Time interaction (F (1,31) = 4.314, *p* = 0.046) for plasma 11-COOH-THC (Figure 4D).

#### 3.2.3 Effects of age on Δ9THC and its metabolites

**FIGURE 5:**
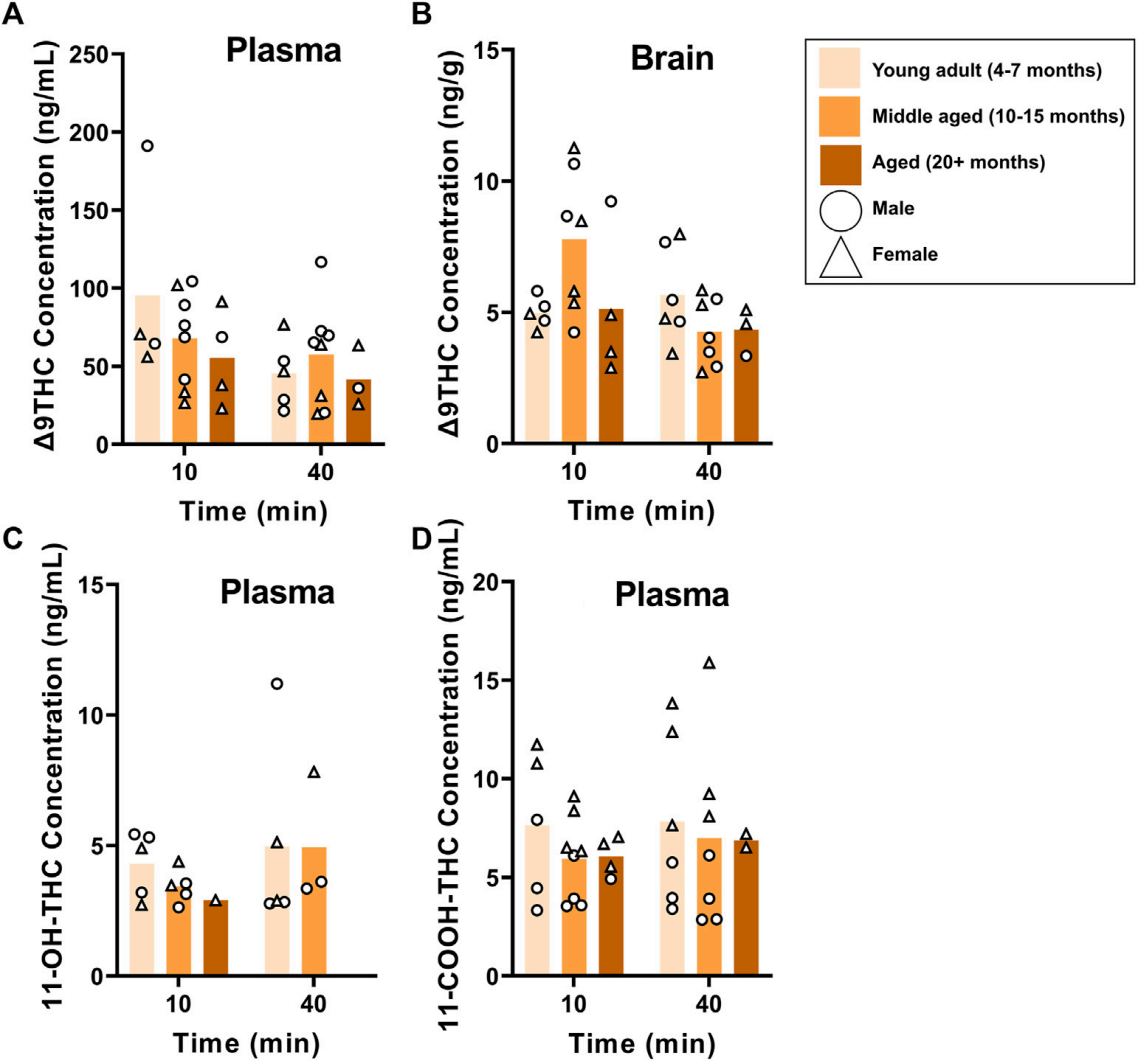
Effects of age on Δ9THC and its metabolite concentrations after smoke exposure in young adult, middle-aged, and advanced age C57BL/6J mice (Experiment 2). There was no main effect of Age on plasma Δ9THC **(A)**, brain Δ9THC **(B)**, or plasma metabolites **(C, D)**. A significant Age × Time interaction in brain Δ9THC concentrations **(B),** however, revealed that middle aged mice had increased concentrations 10 min post-smoke exposure compared to all other age groups at both time points.

B6 and FVB mice were used to assess the effects of age on Δ9THC and metabolite concentrations, as these strains had more than one age group. Plasma and brain Δ9THC and plasma metabolite concentrations in young adult, middle aged, and aged B6 mice following 1 hour of cannabis smoke exposure are shown in Figure 5. For plasma Δ9THC (Figure 5A), a two-way ANOVA (Time x Age) revealed neither main effects of Time (F (1,26) = 3.474, *p* = 0.074) or Age (F (2,26) = 0.769, *p* = 0.474), nor a Time × Age interaction (F (2,26) = 0.987, *p* = 0.386). The same analysis conducted on brain Δ9THC concentrations (Figure 5B) also revealed no main effects of Time (F (1,26) = 2.853, *p* = 0.103) or Age (F (2,26) = 1.090, *p* = 0.351); however, there was a significant Time × Age interaction (F (2,26) = 3.741, *p* = 0.037) with middle aged mice having higher levels at the 10 min time point compared to both young adult and aged mice at both time points. There were no significant main effects of Time or Age, nor a significant Age × Time interaction, for 11-OH-THC and 11-COOH-THC concentrations (Fs < 0.944, ps > 0.348; Figures 5C, D).

**FIGURE 6:**
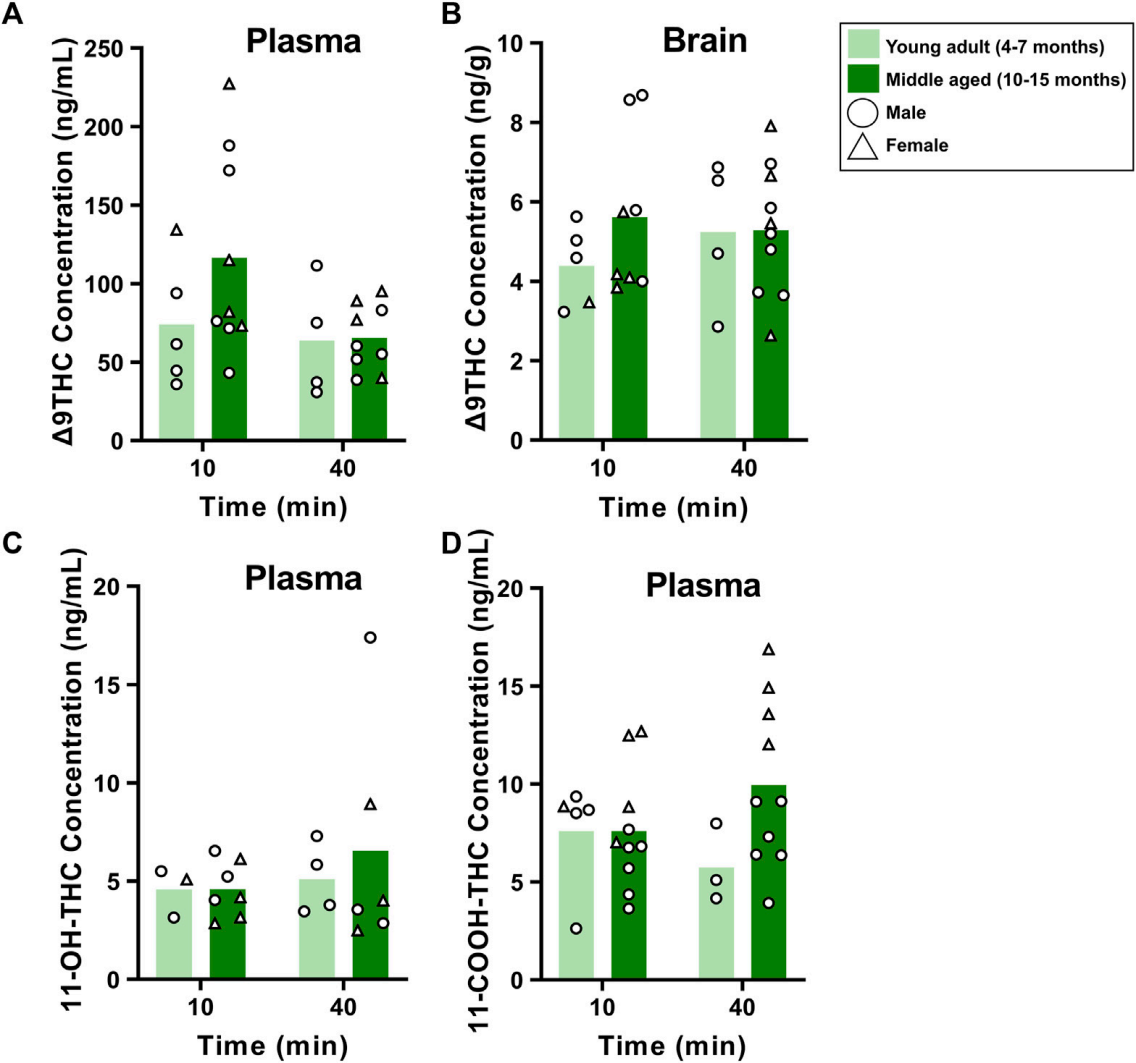
Effects of age on Δ9THC and its metabolite concentrations after smoke exposure in young adult and middle aged FVB mice (Experiment 2). There were no main effects of Age or Age × Time interactions on plasma Δ9THC **(A)**, brain Δ9THC **(B)**, or plasma metabolite concentrations **(C, D)**.

The same analysis in young adult and middle aged FVB mice showed no main effects of Age or Time, nor significant Age × Time interactions, in Δ9THC plasma and brain concentrations (Fs < 2.740, ps > 0.111; Figures 6A, B) or plasma 11-OH-THC and 11-COOH-THC concentrations (Fs < 2.063, ps > 0.164; Figures 6C, D).

### 3.3 Experiment 3. Δ9THC and its metabolites following different durations of smoke exposure

**FIGURE 7:**
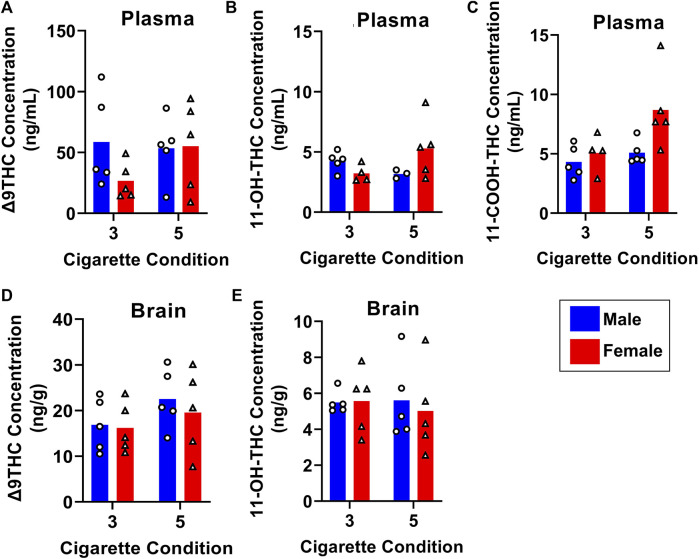
Effects of dose on Δ9THC and its metabolite concentrations in young adult C57BL/6J mice (Experiment 3). Mice were exposed to smoke from burning either 3 or 5 cannabis cigarettes, followed by sample collection 40 min post-smoke exposure. There were no effects of Sex or Dose on Δ9THC and 11-OH-THC in either plasma or brain **(A, B, D, E)**. Females had significantly higher plasma 11-COOH-THC concentrations compared to males, and the 5-cigarette dose yielded significantly higher 11-COOH-THC concentrations in all mice **(C)**.

To determine how Δ9THC and metabolite levels differ as a function of the amount of cannabis smoke exposure, B6 mice were exposed to smoke generated from burning either 3 or 5 cannabis cigarettes, with brain and plasma samples collected 40 min after smoke exposure. Figure 7 displays plasma and brain Δ9THC, 11-OH-THC, and 11-COOH-THC concentrations in males and females in both cigarette conditions. A two-way ANOVA (Cigarette number x Sex) revealed no significant main effects of Cigarette number or Sex, and no significant Cigarette number × Sex interactions, for plasma Δ9THC or plasma 11-OH-THC (Figures 7A, B). Main effects of Cigarette number (F (1, 15) = 5.4,83, *p* = 0.033) and Sex (F (1, 15) = 5.393, *p* = 0.035) were observed for plasma 11-COOH-THC concentrations (Figure 7C), but there was no Cigarette number × Sex interaction (F (1,15) = 2.286, *p* = 0.151). The same analysis conducted on brain Δ9THC and 11-OH-THC concentrations showed no main effects of Cigarette number or Sex, nor were there significant Cigarette number × Sex interactions (Fs < 2.146, ps > 0.162; Figures 7D, E). Brain 11-COOH-THC was undetected in all samples.

## 4 Discussion

The present study assessed the pharmacokinetics of Δ9THC and its two major metabolites, 11-OH-THC and 11-COOH-THC, in both plasma and brain following cannabis smoke exposure. To our knowledge, this is the first evaluation of both plasma and brain concentrations of all three analytes following cannabis smoke exposure in male and female mice. We established a time course of Δ9THC and metabolite concentrations, and assessed the effects of strain, sex, age, and dose on plasma and brain concentrations.

### 4.1 Plasma Δ9THC

Previous studies measuring plasma Δ9THC concentrations following cannabis smoke exposure in male mice found that exposure to smoke from burning 200 mg of cannabis (3.5% THC) results in C_max_ of plasma Δ9THC ranging between 385–402 ng/mL (Lichtman et al., 2001; Wilson et al., 2006). These values are significantly higher than the C_max_ values obtained in Experiment 1 (82.2 ng/mL in males and 47.9 ng/mL in females); however, these prior studies employed a “nose-only” smoke exposure model (mice were restrained such that their nose was in the smoke stream), which could account for the high Δ9THC plasma concentrations. A recent study using a protocol consisting of two smoke exposure sessions with 6 cannabis cigarettes (10%–14% THC) burned in each session resulted in C_max_ of plasma Δ9THC ranging between 125–150 ng/mL in male and female mice (Fantauzzi et al., 2021). These levels are more comparable to those achieved in the present study, particularly since their cannabis cigarettes contained considerably higher Δ9THC content. Similar to the present study, mice in the Fantauzzi et al. study were subjected to whole body smoke exposure, which could account for the lower plasma Δ9THC concentrations compared to studies utilizing nose-only smoke exposure.

One aim of the current study was to evaluate whether the route of cannabis administration (whole-body exposure in freely-moving mice) produced Δ9THC concentrations comparable to those observed in humans. Pharmacokinetic studies of smoked cannabis in humans have shown C_max_ values for plasma Δ9THC ranging from 46.5–76 ng/mL in occasional cannabis users (Toennes et al., 2008; Fabritius et al., 2013; Hartman et al., 2015; Newmeyer et al., 2016; Matheson et al., 2020) and 95–153 ng/mL in heavy cannabis users (Toennes et al., 2008; Fabritius et al., 2013; Newmeyer et al., 2016). Since humans can manipulate smoke topography to achieve higher plasma Δ9THC C_max_ and often use cannabis containing high THC concentrations (e.g., >15%) (NIDA, 2021), animal models require longer exposure to cannabis smoke to achieve similar C_max_ levels. In the current study, a 5-cigarette (1 h) regimen was used to account for lower cannabis THC concentrations (∼6%) and the large percentage (∼50%) of THC destroyed by pyrolysis (Ravula et al., 2019). Moreover, while the freely-moving, whole-body exposure model avoids the restraint stress necessary to implement nose-only exposure (e.g., Lichtman et al., 2001), the amount of exposure achieved is less than in such models and therefore requires a longer duration of smoke inhalation (indeed, the dose of Δ9THC to which mice were exposed in Experiment 1 was estimated at 0.19 mg/kg; Table 3). Nevertheless, the 5-cigarette regimen yielded plasma Δ9THC C_max_ values in mice that fall within those ranges observed in humans, suggesting that it has reasonable translational validity. Additionally, Experiment 3 explored a shorter duration of smoke exposure (∼36 min) using 3 cigarettes, and found that it produced plasma and brain Δ9THC concentrations comparable to the 5 cigarette regimen.

There are inconsistencies in regard to sex differences in Δ9THC plasma concentrations in humans, with some studies noting significantly lower concentrations in females compared to males (Matheson et al., 2020) and other studies finding no sex differences (Sholler et al., 2021; Arkell et al., 2022). Similarly, in the current study, Experiment 1 found that females achieved C_max_ values approximately half of those in males, and at a later time point compared to males, whereas in Experiment 2 there were no sex differences at the 10-min time point in young adult mice collapsed across strain. Notably, however, while there were no significant Sex × Time interactions or main effects of Sex in young adult mice in each separate strain in Experiment 2, B6 males had numerically higher levels than B6 females 10 min post-smoke exposure, whereas there was an opposite trend in FVB and SW mice, with females having higher levels than males at the 10 min time point. These contrasting trends may have contributed to the absence of overall sex differences when all mice were collapsed across strains. Aside from the Sex × Time interaction revealed in Experiment 1, there were no significant strain, sex, age, no dose differences in plasma Δ9THC concentrations in the present study.

### 4.2 Brain Δ9THC

In Experiment 1, peak brain Δ9THC concentrations were achieved in male (21.2 ng/g) and female (23.2 ng/g) mice 20 and 40 min following smoke exposure, respectively. This delay relative to peak plasma levels was expected, as it takes time for the drug to distribute into tissues, and is consistent with a previous study of vaporized Δ9THC in rats that showed peak brain concentrations achieved 15 and 30 min following THC vapor exposure in males and females, respectively (Baglot et al., 2021). Pharmacokinetic studies of cannabis smoke exposure in mice show Δ9THC concentrations of 136–400 ng/g in brains collected 20 min following smoke exposure (Wilson et al., 2006; Poklis et al., 2010); however, these studies used nose-only smoke exposure in which considerably higher plasma Δ9THC were obtained. In studies of vaporized THC in adult rats, peak Δ9THC brain concentrations ranged between 110–125 ng/g when rats were exposed to 100 mg/mL and 200 mg/mL Δ9THC (Hložek et al., 2017; Baglot et al., 2021). Although the peak values obtained in Experiment 1 fall below the ranges of prior rodent studies, they are consistent with a study conducted in postmortem human brain tissues from 11 cases positive for cannabinoids, which reported Δ9THC brain concentrations ranging between 3.71–43.6 ng/g (Saenz et al., 2017).

There were no sex differences in brain Δ9THC concentrations in any of the three experiments; however, Experiment 2 revealed that strain and age can influence brain Δ9THC levels. This experiment revealed a main effect of strain on Δ9THC brain concentrations, with 129 mice achieving higher levels compared to FVB mice. One possible explanation for this result is that FVB mice are more active than 129 mice (Bothe et al., 2004; Wilson et al., 2006), and some studies suggest that physical activity promotes clearance of Δ9THC from fatty tissues back into circulation, where it can travel to the liver and be metabolized (Wong et al., 2013; Wong et al., 2014). Indeed, FVB mice had numerically higher levels of the secondary Δ9THC metabolite 11-COOH-THC compared to 129 mice, which is consistent with this interpretation. Additionally, in the B6 strain, middle-aged mice (10–15 months old) had the highest brain Δ9THC concentrations 10 min following smoke exposure compared to the other age groups at both time points. Aging is associated with decreases in water content and increases in fat content, and thus the distribution volume of lipophilic compounds such as Δ9THC is generally increased (Turnheim, 2003). Although less is known about Δ9THC accumulation and clearance in the brain specifically, it is safe to hypothesize that these increases in fat content, along with reduced liver function and physical activity, can affect Δ9THC concentrations in the brain. Somewhat surprisingly, greater levels of brain Δ9THC were not detected in mice of advanced age (20+ months old), although the smaller sample size at this age precluded robust analysis of age differences.

### 4.3 Δ9THC metabolites

Δ9THC is mainly hydroxylated by CYP450 2C9, 2C19, and 3A4 enzymes, leading to production of the metabolite 11-OH-THC (other minor metabolites such as 8β-OH-THC can also be formed, depending on the site of hydroxylation) (Matsunaga et al., 1995; Yamamoto et al., 1995). 11-OH-THC is oxidized to the secondary metabolite, 11-COOH-THC, which, unlike Δ9THC and 11-OH-THC, does not have affinity for the CB1 receptor and is non-psychoactive (Andrenyak et al., 2017). Due to its rapid oxidation into the secondary metabolite, 11-OH-THC does not accumulate at high levels in either plasma or brain (Huestis, 2007). The results of Experiment 1 are consistent with these properties, as plasma and brain concentrations of 11-OH-THC were below the LLOQ for almost all samples obtained after the 40-min time point. Previous work showed that 11-OH-THC plasma concentrations are approximately 6%–10% of plasma Δ9THC concentrations 45 min after the start of smoking (Wall and Perez-Reyes, 1981; Huestis et al., 1992; Karschner et al., 2009), meaning that approximately 2.9–8.2 ng/mL concentrations of plasma 11-OH-THC would be expected in the present study based on peak plasma Δ9THC concentrations of 48–82 ng/mL. Indeed, a majority of the quantifiable samples had 11-OH-THC values in that range. Rat pharmacokinetic studies show peak plasma concentrations ranging between 2–25 ng/mL for 11-OH-THC (Baglot et al., 2021; Ruiz et al., 2021b) and human studies range between 2–14.2 ng/mL (Fabritius et al., 2013; Hartman et al., 2015; Lee et al., 2015; Newmeyer et al., 2016; Matheson et al., 2020; Sholler et al., 2021; Arkell et al., 2022), which also matches the results of the present study. Samples from Experiment 2 and 3 revealed no sex differences in plasma or brain 11-OH-THC concentrations. Although there were no main effects or interactions involving strain or age on plasma 11-OH-THC concentrations in Experiment 2, unequal distribution of mouse demographics prevented full comparisons across all strains and age groups.

Despite the fact that sex differences were not observed in 11-OH-THC plasma concentrations, all three experiments revealed robust sex differences in 11-COOH-THC plasma concentrations, with females achieving higher concentrations than males. Inhaled cannabis pharmacokinetic studies in rats have shown the same pattern (Baglot et al., 2021; Ruiz et al., 2021b); however, metabolite analyses in mouse studies utilizing cannabis smoke exposure have been largely unexplored. Human pharmacokinetic studies have not found consistent sex differences in 11-COOH-THC concentrations, with reports of either lower concentrations in females compared to males (Matheson et al., 2020; Sholler et al., 2021) or no sex differences (Arkell et al., 2022). The sex differences supported by our study may point to species-specific differences in Δ9THC metabolism. One possible explanation for these results concerns differences in cytochrome P450 enzyme activity. Early studies showed that cytochrome P450 enzymes in female rat liver primarily metabolize Δ9THC into 11-OH-THC, whereas males produce 11-OH-THC along with multiple other primarily inactive metabolites due to regioselectivity differences in male and female enzymes (Narimatsu et al., 1991; Yamamoto et al., 1995). Given that females primarily metabolize Δ9THC into 11-OH-THC, which then further oxidizes into 11-COOH-THC, these sex differences in the secondary metabolite fall in line with the differences in enzyme activity. Although this idea would predict sex differences in plasma 11-OH-THC concentrations as well, these differences might not be captured due to the rapid oxidation of 11-OH-THC, whereas 11-COOH-THC accumulates more readily due to its slower clearance. This difference is important to consider, particularly in research assessing effects of cannabis on inflammation and neurodegenerative conditions associated with neuroinflammation such as Alzheimer’s disease, as 11-COOH-THC remains in the body for extended periods of time (Grotenhermen, 2003; Sharma et al., 2012) and has been suggested to have anti-inflammatory properties (Ujváry and Grotenhermen, 2014). In humans, heavy/frequent cannabis smokers have peak 11-COOH-THC concentrations between 45–155 ng/mL (Fabritius et al., 2013; Lee et al., 2015; Newmeyer et al., 2016), whereas occasional cannabis users have ranges between 7.2 and 15 ng/mL (Fabritius et al., 2013; Newmeyer et al., 2016). Peak values for mice in the current study were within the range of occasional cannabis users, consistent with the fact that they were naïve to cannabis prior to exposure. As 11-COOH-THC concentrations accumulate in frequent users, it will be important in future studies to evaluate 11-COOH-THC concentrations following chronic cannabis smoke exposure. There were no differences in plasma 11-COOH-THC concentrations as a function of mouse strain or age. Finally, 11-COOH-THC concentrations in brain samples were below the LLOQ in all experiments, which agrees with the fact that the compound is less lipophilic than Δ9THC and 11-OH-THC and has less propensity to accumulate in brain tissue (Huestis, 2007).

### 4.4 Limitations and conclusions

Limitations of this study include starting sample collection 10 min following smoke exposure. Although this timepoint was the earliest possible given the logistics of sample collection, it may have reduced C_max_ values for Δ9THC (as well as levels of 11-OH-THC) since peak values can be achieved as early as 3 min following exposure (Grotenhermen, 2003). Additionally, in Experiment 2, more balanced demographics of mice could have allowed for strain and age comparisons with greater statistical power. An area of future work could include a similar full evaluation of Δ9THC and its metabolites through an oral route of administration, especially if translating to medicinal use since smoking has numerous adverse health effects.

In conclusion, the current data fill gaps in pharmacokinetic studies of inhaled cannabis/cannabinoids in mice by including both sexes and evaluating both 11-OH-THC and 11-COOH-THC in plasma and brain. In particular, the results show that both age and mouse strain can influence Δ9THC pharmacokinetics, which emphasizes the importance of taking these variables into consideration when evaluating cannabinoid pharmacology in mice. With the growth in research on cannabis therapeutics for neurodegenerative conditions such as Alzheimer’s disease, studies utilizing transgenic mice in particular need to consider both genetic background and the age of the animal, especially if modelling a disease not typically occurring in young adults. Additionally, there are robust sex differences in Δ9THC metabolism, which is important to consider in cannabinoid studies since these differences are not consistent with some findings in humans and thus may be specific to rodents. Lastly, a 3-cigarette dose resulted in plasma and brain Δ9THC concentrations comparable to the 5-cigarette dose, which suggests that the lower dose should be sufficient when studying the effects of cannabis in mice.

## Data Availability

The raw data supporting the conclusion of this article will be made available by the authors, without undue reservation.
