# Supplementary material for: Pharmacokinetics of delta-9-tetrahydrocannabinol following acute cannabis smoke exposure in mice; effects of sex, age, and strain
Source: Front Pharmacol. 2023 Aug 28;14:1227220. doi: 10.3389/fphar.2023.1227220 (PMC10493391; doi:10.3389/fphar.2023.1227220)
Supplement: Supplementary file 1 [file DataSheet1.docx]

Pharmacokinetics of delta-9-tetrahydrocannabinol following acute cannabis smoke exposure in mice; effects of sex, age, and strain

Emely A Gazarov^1,2^, Sabrina Zequeira^1^, Alexandria S Senetra^3^, John Howard^1^, Abhisheak Sharma^3,5^, Christopher R McCurdy^3,4,5^, Jada Lewis^1^, Jennifer L Bizon^1,2,5^, Barry Setlow^1,2,5*^

^1^University of Florida, Department of Neuroscience, Gainesville, FL, USA

^2^University of Florida, Department of Psychiatry, Gainesville, FL, USA

^3^University of Florida, Department of Pharmaceutics, Gainesville, FL, USA

^4^University of Florida, Department of Medicinal Chemistry, Gainesville, FL, USA

^5^University of Florida, Center for Addiction Research and Education, Gainesville, FL, USA


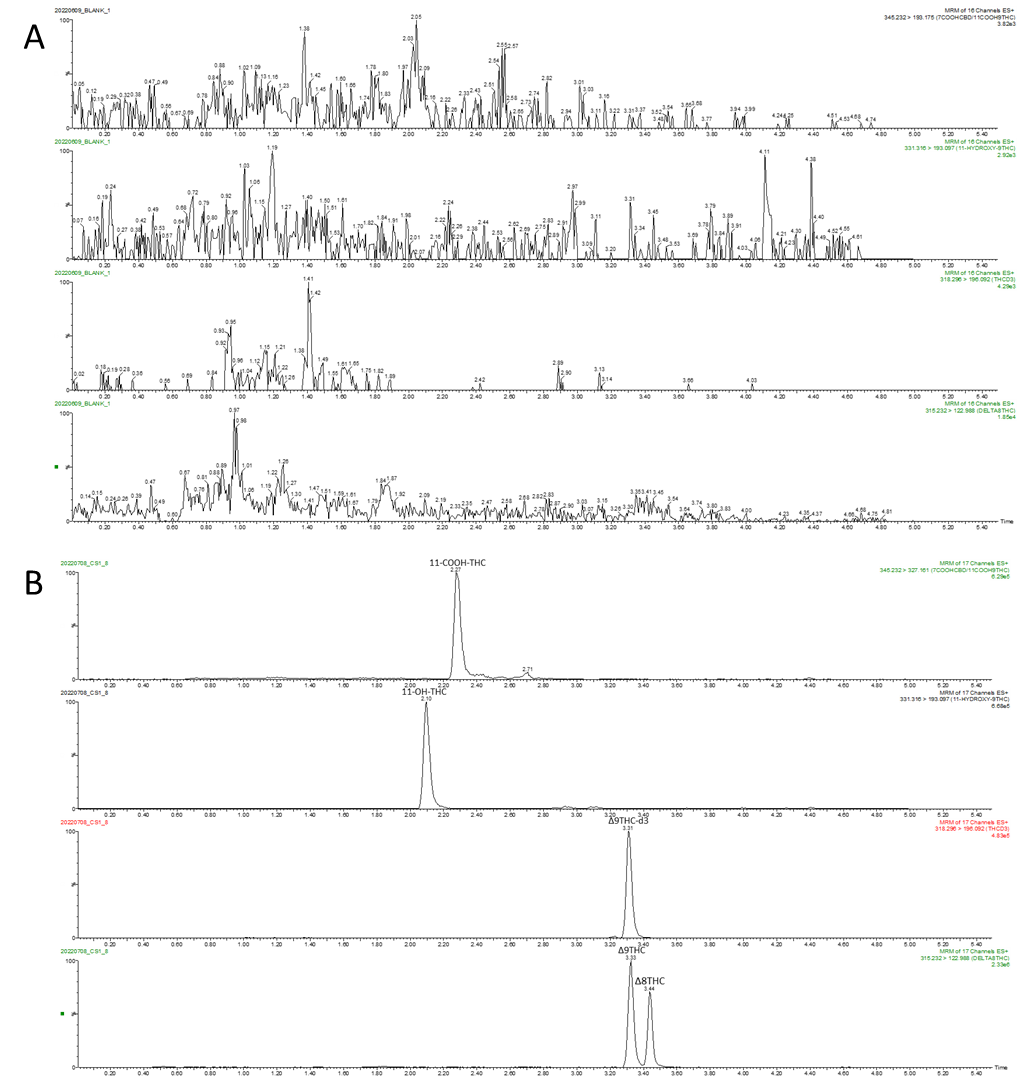


Supplemental Figure 1: Representative chromatograms for Δ8THC, Δ9THC, Δ9THC-d3, 11-OH-THC, and 11-COOH-THC (A) blank plasma; (B) analytes spike in blank plasma (250 ng/mL) in positive ionization mode.

| Supplemental Table 1: Accuracy and precision of the assay in mouse plasma and brain homogenate | | | | | | | | |
| --- | --- | --- | --- | --- | --- | --- | --- | --- |
| Δ8THC | Plasma | | | | Brain Homogenate | | | |
|  | Intra-day | | Inter-day | | Intra-day | | Inter-day | |
| Concentration (ng/mL) | Precision (%RSD) | Accuracy (% Bias) | Precision (%RSD) | Accuracy (% Bias) | Precision (%RSD) | Accuracy (% Bias) | Precision (%RSD) | Accuracy (% Bias) |
| 2.50 | 3.96 | 16.40 | 18.79 | 1.07 | 2.72 | -3.87 | 11.68 | 0.93 |
| 7.50 | 3.32 | -12.89 | 8.50 | -4.27 | 7.90 | 3.38 | 2.30 | -3.82 |
| 125.00 | 3.05 | 2.39 | 0.97 | 6.19 | 4.13 | -4.55 | 3.53 | -3.02 |
| 200.00 | 6.01 | 1.97 | 4.06 | 6.16 | 2.30 | -7.80 | 5.72 | -6.93 |
| Δ9THC | Plasma | | | | Brain Homogenate | | | |
|  | Intra-day | | Inter-day | | Intra-day | | Inter-day | |
| Concentration (ng/mL) | Precision (%RSD) | Accuracy (% Bias) | Precision (%RSD) | Accuracy (% Bias) | Precision (%RSD) | Accuracy (% Bias) | Precision (%RSD) | Accuracy (% Bias) |
| 2.50 | 4.73 | 13.47 | 7.33 | 11.47 | 6.83 | -4.00 | 4.98 | 8.53 |
| 7.50 | 6.56 | 4.31 | 5.51 | -0.49 | 9.37 | -0.27 | 9.60 | 0.58 |
| 125.00 | 3.96 | 4.70 | 6.37 | 3.42 | 6.26 | -5.59 | 10.38 | -0.38 |
| 200.00 | 1.59 | 7.80 | 4.97 | 6.05 | 1.87 | -1.30 | 4.91 | 2.09 |
| 11-OH-THC | Plasma | | | | Brain Homogenate | | | |
|  | Intra-day | | Inter-day | | Intra-day | | Inter-day | |
| Concentration (ng/mL) | Precision (%RSD) | Accuracy (% Bias) | Precision (%RSD) | Accuracy (% Bias) | Precision (%RSD) | Accuracy (% Bias) | Precision (%RSD) | Accuracy (% Bias) |
| 2.50 | 1.53 | 12.80 | 5.81 | 6.80 | 10.50 | 10.67 | 4.05 | -9.47 |
| 7.50 | 6.33 | 6.67 | 3.94 | 4.49 | 10.35 | -0.13 | 4.82 | 3.82 |
| 125.00 | 3.70 | 5.93 | 6.35 | 3.15 | 6.28 | -8.12 | 7.33 | -3.29 |
| 200.00 | 3.34 | 9.84 | 6.08 | 6.74 | 2.56 | -4.57 | 2.16 | -0.29 |
| 11-COOH-THC | Plasma | | | | Brain Homogenate | | | |
|  | Intra-day | | Inter-day | | Intra-day | | Inter-day | |
| Concentration (ng/mL) | Precision (%RSD) | Accuracy (% Bias) | Precision (%RSD) | Accuracy (% Bias) | Precision (%RSD) | Accuracy (% Bias) | Precision (%RSD) | Accuracy (% Bias) |
| 2.50 | 4.36 | 8.80 | 6.46 | 5.33 | 14.24 | 1.60 | 5.25 | 3.33 |
| 7.50 | 3.69 | 7.16 | 3.27 | 4.62 | 12.74 | 1.24 | 7.24 | -6.40 |
| 125.00 | 2.61 | 7.60 | 5.42 | 5.13 | 8.14 | -3.67 | 7.63 | -4.27 |
| 200.00 | 0.25 | 10.99 | 3.97 | -3.95 | 1.18 | -2.05 | 5.67 | -6.10 |
